# *Natronomonas salsuginis* sp. nov., a New Inhabitant of a Marine Solar Saltern

**DOI:** 10.3390/microorganisms8040605

**Published:** 2020-04-21

**Authors:** Ana Durán-Viseras, Cristina Sánchez-Porro, Antonio Ventosa

**Affiliations:** Department of Microbiology and Parasitology, Faculty of Pharmacy, University of Sevilla, 41012 Sevilla, Spain; anaduran@us.es (A.D.-V.); sanpor@us.es (C.S.-P.)

**Keywords:** Haloarchaea, hypersaline habitats, taxonomy, *Natronomonas*, rhodopsins

## Abstract

A halophilic archaeon, strain F20-122^T^, was isolated from a marine saltern of Isla Bacuta (Huelva, Spain). Cells were Gram-stain-negative, aerobic, and coccoid in morphology. It grew at 25–50 °C (optimum 37 °C), pH 6.5–9.0 (optimum pH 8.0), and 10–30% (*w*/*v*) total salts (optimum 25% salts). The phylogenetic analyses based on the 16S rRNA and *rpoB’* genes showed its affiliation with the genus *Natronomonas* and suggested its placement as a new species within this genus. The in silico DNA–DNA hybridization (DDH) and average nucleotide identity (ANI) analyses of this strain against closely related species supported its placement in a new taxon. The DNA G + C content of this isolate was 63.0 mol%. The polar lipids of strain F20-122^T^ were phosphatidylglycerol phosphate methyl ester (PGP-Me), phosphatidylglycerol (PG), and phosphatidylglycerol sulfate (PGS). Traces of biphosphatidylglycerol (BPG) and other minor phospholipids and unidentified glycolipids were also present. Based on the phylogenetic, genomic, phenotypic, and chemotaxonomic characterization, we propose strain F20-122^T^ (= CCM 8891^T^ = CECT 9564^T^ = JCM 33320^T^) as the type strain of a new species within the genus *Natronomonas*, with the name *Natronomonas salsuginis* sp. nov. Rhodopsin-like sequence analysis of strain F20-122^T^ revealed the presence of haloarchaeal proton pumps, suggesting a light-mediated ATP synthesis for this strain and a maximum wavelength absorption in the green spectrum.

## 1. Introduction

The genus *Natronomonas* belongs to the family *Haloarculaceae*, within the order *Halobacteriales*, class *Halobacteria* in the phylum *Euryarchaeota*. The type species of the genus is *Natronomonas pharaonis*, which was isolated from alkaline brines in Egypt [1]. This species was first described as *Halobacterium pharaonis* [1]. After that, it was transferred to the genus *Natronobacterium* as *Natronobacterium pharaonis* [2], and finally, Kamekura et al. [3] proposed the transfer of *Natronobacterium pharaonis* to the new genus *Natronomonas* based on its 16S rRNA sequence similarity. At the time of writing, the genus *Natronomonas* comprises two other described species [4]—*Natronomonas moolapensis* [5] and *Natronomonas gomsonensis* [6]—which were isolated from marine salterns of Australia and Korea, respectively. 

One of the particular features of this genus is that it comprises alkaliphilic and nonalkaliphilic species. While *Natronomonas moolapensis* and *Natronomonas gomsonensis* are nonalkaliphilic, the type species of the genus, *Natronomonas pharaonis*, is able to grow between pH values of 8.0 and 11.0, with the optimum at pH 8.5–9.0, being the first alkaliphilic extreme halophile described. The genus *Natronomonas* comprises motile and Gram-stain-negative species with a wide variety of cell morphologies, from short rods to coccoid-shaped or flat tetragonal shapes [5,6]. Other characteristics of species of this genus are their red-pigmented colonies and an aerobic and chemo-organotrophic metabolism [5,6]. The species of the genus *Natronomonas* are halophilic, which require at least 2 M NaCl for growth. The major polar lipids are phosphatidylglycerol, phosphatidylglycerophosphate methyl ester, and phosphatidic acid. Undetermined phospholipids or glycolipid may exist and phytanyl-sesterpanyl moieties (C_20_C_25_) are also present [5]. The DNA G + C content of the strains ranged from 61.8 to 64.3 mol% [3,5,6].

The genus *Natronomonas* is one of the oldest and best known haloarchaeal genera. Their rhodopsins and their applications have been deeply studied. Besides, several studies have focused on the biotechnological potential of its enzymes [7,8,9,10,11,12,13,14,15]. On the other hand, several culture-independent studies have also shown the presence of members of the genus *Natronomonas* in different environments from widely separated sites, such as hypersaline and alkaline lakes, solar salterns, saline–alkaline soils, and sediments or oil fields [16,17,18,19,20,21,22,23,24,25,26,27,28,29,30,31], supporting the widespread distribution of species of this genus.

During the course of the study of the prokaryotic diversity inhabiting various hypersaline environments of southwest Spain, a new haloarchaeon, designated strain F20-122^T^, was isolated in pure culture. Based on the 16S rRNA gene sequence analysis, this strain was phylogenetically related to species of the genus *Natronomonas*. In this paper, we describe the isolation and taxogenomic characterization of this archaeon and propose it as a new species of the genus *Natronomonas*, for which we propose the name *Natronomonas salsuginis* sp. nov.

## 2. Materials and Methods 

### 2.1. Isolation, Culture, and Preservation of Haloarchaeal Strain

Strain F20-122^T^ was isolated from a water sample obtained in June 2016 from a pond of a marine saltern located in Isla Bacuta, Huelva, southwest coast of Spain (37°15’N 6°58′W). At the time of sampling, the salinity of the water was 17% (*w*/*v*) and the pH was 7.8. Samples were collected in sterile containers, transported to the laboratory, and plated under sterile conditions. Plates were incubated at 37 °C for up to 3 months. Strain F20-122^T^ was isolated in pure culture after three successive cultivations on R2A medium (Difco) supplemented with 25% seawater salts solution, with the following composition (g L^−1^): NaCl, 195; MgCl_2_.6H_2_O, 32.5; MgSO_4_.7H_2_O, 50.8; CaCl_2_, 0.83; KCl, 5.0; NaHCO_3_, 0.21; NaBr, 0.58. The pH was adjusted to 7.5 with 1 M KOH. Purified agar (2%; Oxoid) was used as a solidifying agent. Strain F20-122^T^ was routinely grown in the same medium of isolation (designated R2A25%) and incubated aerobically at 37 °C for 14 days, using a rotary shaker for growth in liquid medium. Cultures were maintained at −80 °C in R2A25% medium containing 25% (*v*/*v*) glycerol for long-time preservation. The type strains *Natronomonas moolapensis* CECT 7526^T^ and *Natronomonas pharaonis* CECT 4578^T^ were used as reference strains for comparative purposes in our study.

### 2.2. DNA Extraction, Purification, and Sequencing

Using the method described by Marmur [32], the genomic DNA from strain F20-122^T^ was extracted and purified for 16S rRNA and *rpoB´* gene analysis and for genome sequencing. The quality of the DNA was checked by (1%) agarose gel electrophoresis. DNA quantification was determined by spectrophotometry (DeNovix DS-11 FX, DeNovix Technologies, Wilmington, Delaware, USA) and fluorometry (Qubit 3.0 Fluorometer, Thermofisher Scientific, USA). The 16S rRNA and *rpoB´* genes were amplified by PCR [33] with the universal primers ArchF and ArchR [34,35] and the primers designed by Fullmer et al. [36], respectively. The PCR products were sequenced by StabVida (Oeiras, Portugal) using the Sanger method and the same primers. For the 16S rRNA gene sequencing, primers 16RB36 (GGA CTA CCA GGG TAT CTA) and 16RD34 (GGT CTC GCT CGT TGC CTG) were also used. Sequencing reactions were carried out using a BigDye terminator kit version 3.1 from Applied Biosystems. A draft genome sequence of strain F20-122^T^ was also determined in this study using a whole-genome shotgun strategy. After the DNA quality control, a library was constructed using the Kappa HyperPrep library preparation kit. The generated DNA libraries were sequenced in the lllumina Hiseq 4000 platform, using 150 bp paired-end sequencing reads (StabVida, Oeiras, Portugal).

### 2.3. Phylogenetic Analyses

The 16S rRNA and *rpoB’* gene sequences of strain F20-122^T^ were aligned with ChromasPro (Technelysium Pty Ltd.) software version 1.5 and deposited in GenBank/EMBL/DDBJ under the accession numbers MH424601 and MH454090, respectively. The identification of phylogenetic neighbors and the calculation of pairwise 16S rRNA and *rpoB’* gene sequence similarities were achieved using the EzBioCloud tool [37] and BLAST [38], respectively. The 16S rRNA gene sequence analysis and phylogenetic tree reconstructions were performed with the ARB software package [39]. Phylogenetic trees were inferred using three different methods—maximum-parsimony [40], neighbor-joining [41], and maximum-likelihood [42] algorithms—integrated in the ARB software. A bootstrap analysis (1000 replications) was performed to evaluate the robustness of the phylogenetic trees [43]. For the construction of the trees based on the *rpoB’* gene sequence, MEGA 6.0 software was used with the neighbor-joining and maximum-likelihood algorithms [44]. The 16S rRNA and *rpoB´* gene sequences used for phylogenetic comparisons were obtained from the GenBank database. For the phylogenomic core genome analysis reconstruction, all-versus-all BLAST search [38] was used for comparisons of all predicted protein-coding genes annotated from each available genome. MUSCLE [45] was used for the individual alignment of the core orthologous genes with diagonal optimization and adjusting to the maximum number of iterations (default values for the other parameters). The phylogenomic tree was reconstructed by using MEGA 6.0 software (neighbor-joining method with Jukes–Cantor correction) [44]. For the Venn diagram representation, the online software InteractiVenn was used [46].

### 2.4. Genome Assembly, Annotation, and Determination of Genomic Parameters

For the de novo assembly of the reads, Spades v.3.12.0. [47] was used. CheckM v1.0.5 [48] and Quast v2.3 [49] were utilized to assess the quality of the final contigs. The genome sequence of strain F20-122^T^ was annotated using the NCBI Prokaryotic Genome Annotation Pipeline (PGAP) [50] and deposited in GenBank/EMBL/DDBJ under the accession number QKNX00000000.

The genomic parameters average nucleotide identity (ANI) and in silico DNA–DNA hybridization (DDH) between the genome of the strain F20-122^T^ and the genomes of *Natronomonas pharaonis* DSM 2160^T^ (GCF_000026045) and *Natronomonas moolapensis* 8.8.11^T^ (GCF_000591055) were calculated by using OAT software v0.93.1 [51] and the Genome-to-Genome Distance Calculator (GGDC) [52] website, using formula 2 [53], respectively.

### 2.5. Phenotypic Characterization

Phenotypic tests were performed according to the proposed minimal standards for the description of novel taxa in the class *Halobacteria* [54]. *Natronomonas moolapensis* CECT 7526^T^ was used in this study for comparison as a reference strain. *Natronomonas pharaonis* CECT 4578^T^, the type species of the genus, was not included on the phenotypic comparison because it grows at a markedly different pH compared with strain F20-122^T^.

Strain F20-122^T^ was grown on R2A25% medium on a shaking incubator at 200 rpm for the determination of cellular morphology and motility. The cells were examined by light microscopy under a phase-contrast microscope (Olympus BX41). Gram staining was carried out using acetic-acid-fixed samples as described by Dussault [55]. For anaerobic tests, R2A25% plates supplemented with 4% L-arginine, 10% DMSO, and 3% KNO_3_, respectively, were inoculated and incubated at 37 °C in an anaerobic chamber (Oxoid) for 21 days. The morphology of the colonies of strain F20-122^T^, their size, and pigmentation were observed on R2A25% medium up to 14 days of aerobic incubation at 37 °C. The range and optimal conditions of salinity for growth was determined by using R2A liquid medium supplemented with 0.9, 3, 5, 10, 15, 20, 25, and 30% (*w*/*v*) total salts. In order to determine the pH range (and optimum) for growth of strain F20-122^T^, the isolate was cultured under the optimal salt concentration conditions, adjusting the medium to pH 5.0, 6.0, 6.5, 7.0, 7.5, 8.0, 8.5, 9.0, and 10.0 with the addition of the appropriate buffers [56]. The optimal and range of temperature were determined by incubating strain F20-122^T^ under the optimal salt concentration and pH conditions, at temperatures of 4, 15, 20, 25, 30, 37, 40, 45, 50, 55, and 60 °C. Growth rates were determined by monitoring the increase in the optical density at 600 nm. 

Catalase activity was determined by adding a 3% (*w*/*v*) H_2_O_2_ solution to colonies on R2A25% medium. Oxidase activity was examined with 1% (*v*/*v*) tetramethyl-p-phenylenediamine [57]. All biochemical tests were carried out in R2A25% medium (pH 7.5) at 37 °C, unless otherwise stated. Hydrolysis of aesculin, gelatin, or starch was carried out as described by Barrow and Feltham [58]. Tests for indole production from tryptophan and urease test were performed as described by Gerhardt et al. [59]. Voges–Proskauer and methyl red tests were determined as described by Oren et al. [54]. The reduction of nitrate and nitrite were detected by using sulfanilic acid and α-naphthylamine reagents [60]. H_2_S formation was determined following the methodology described by Clarke [61]. Citrate utilization was determined on Simmons’ citrate medium supplemented with a 25% seawater salts solution (SW25) [54]. Acid production from carbohydrates was determined using a modified phenol red base medium prepared with SW25 solution supplemented with 0.5% (*w/v*) yeast extract (Difco) and 1% (*w/v*) of the carbohydrate [54]. To assess the utilization of sole carbon and energy sources, 1% (*w*/*v*) of different carbohydrates, alcohols, amino acids, and organic acids substrates were added individually in SW25 solution containing 0.05% (*w*/*v*) yeast extract (Difco) [62]. Substrates were added as filter-sterilized solutions to give a final concentration of 1 g L^−1^ for organic acids and amino acids, and of 2 g L^−1^ for carbohydrates and alcohols. 

### 2.6. Chemotaxonomic Analysis

Polar lipids of strain F20-122^T^, *Natronomonas pharaonis* CECT 4578^T^, and *Natronomonas moolapensis* CECT 7526^T^ were obtained from biomass cultured in medium R2A25% at 37 °C for 14 days for strain F20-122^T^ and *Natronomonas moolapensis* CECT 7526^T^ and from biomass cultured in the alkaline medium for *Natronomonas pharaonis* CECT 4578^T^ [1]. *Halobacterium salinarum* DSM 3754^T^ and *Halorubrum saccharovorum* DSM 1137^T^ were used as reference species for polar lipid characterization. Polar lipids were extracted with chloroform/methanol following the method for extraction of membrane polar lipids of halophilic archaea described previously by Corcelli and Lobasso [63]. Total lipid extracts were analyzed by high-performance thin layer chromatography (HPTLC), using HPTLC silica gel 60 plates crystal back (10,620 cm; Merck art. 5626); the plates were developed in the solvent system chloroform/methanol/90% acetic acid (65:4:35) [64]. To detect all polar lipids, the plate was sprayed with 5% sulfuric acid in water and charred by heating at 160 °C [65]. To identify phospholipids, another HPTLC was performed and sprayed with molybdenum blue reagent, which selectively stains phospholipids.

### 2.7. Rhodopsin Analysis

Hmmsearch [66] and a profile hidden Markov model (HMM) of the bacteriorhodopsin-like protein family (Pfam accession: PF01036) were used to explore the presence of rhodopsin-like sequences in the genomes of strain F20-122^T^, *Natronomonas pharaonis* DSM 2160^T^, and *Natronomonas moolapensis* 8.8.11^T^. To align the identified sequences and a curated database based on type-1 rhodopsins, MAFFT with the L-INS-i accuracy model [67] was employed. For the rhodopsin tree reconstruction, FastTree2 software [68] (maximum-likelihood algorithm and 100 bootstrap replicates) was used.

## 3. Results and Discussion

### 3.1. Phylogenetic Analyses

From a study of prokaryotic diversity in different hypersaline habitats of southwest Spain, an extensive collection of haloarchaeal strains were recovered, including strain F20-122^T^, which was isolated in pure culture and selected for further analyses.

**Figure 1:**
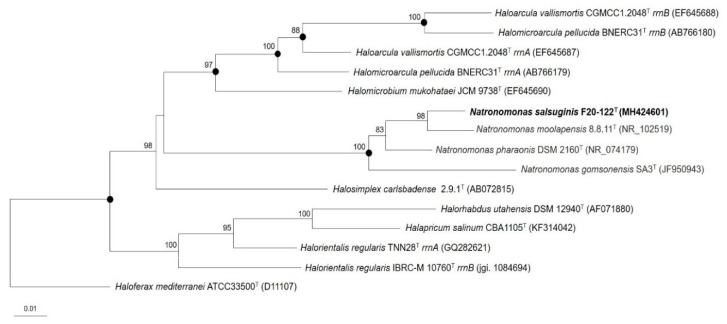
Maximum-parsimony phylogenetic tree reconstruction based on the 16S rRNA gene sequence comparison showing the phylogenetic position of strain F20-122^T^ and the closely related species of the genus *Natronomonas* and other related genera. Sequence accession numbers are shown in parentheses. Bootstrap values higher than 70% are indicated at branch points. Filled circles indicate that the corresponding nodes were also obtained in the trees generated with the neighbor-joining and maximum-likelihood algorithms. The species *Haloferax mediterranei* ATCC 33500^T^ was used as an outgroup. Bar, 0.01 substitutions per nucleotide position.

The almost-complete 16S rRNA gene sequence analysis of strain F20-122^T^ (1,400 bp) showed that it was closely related to members of the genus *Natronomonas*. The highest sequence similarity values were to the 16S rRNA genes of *Natronomonas moolapensis* 8.8.11^T^ (97.7%), *Natronomonas pharaonis* DSM 2160^T^ (97.4%), and *Natronomonas gomsonensis* SA3^T^ (96.1%). The 16S rRNA gene sequence similarity with species of other genera, such as *Salinirubellus* or *Halomarina*, was always equal or lower than 90.7%. The 16S-rRNA-based phylogenetic tree constructed by maximum parsimony (Figure 1) showed that strain F20-122^T^ clustered with the three species of the genus *Natronomonas* but was placed in an independent branch with a bootstrap value of 100%. This topology suggests that the new strain F20-122^T^ could constitute a new member of the genus *Natronomonas*. Trees with similar topology were obtained using the neighbor-joining or maximum-likelihood algorithms (Figure 1). 

**Figure 2:**
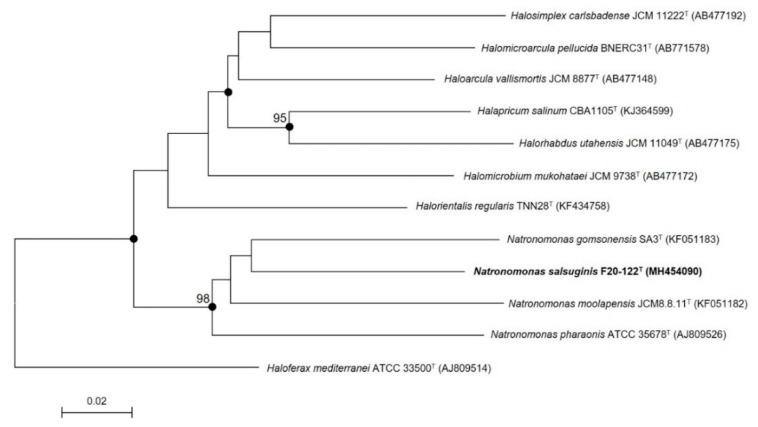
Phylogenetic tree reconstruction based on the *rpoB’* gene of strain F20-122^T^ and related species based on neighbor-joining algorithm. Sequence accession numbers are shown in parentheses. Bootstrap values higher than 70% are indicated at branch points. Filled circles indicate that the corresponding nodes were also obtained in the trees generated with the neighbor-joining and maximum-likelihood algorithms. The species *Haloferax mediterranei* ATCC 3350^T^ was used as an outgroup. Bar, 0.02 substitutions per nucleotide position.

To avoid the limitations of using only the 16S rRNA gene [69,70], other phylogenetic approaches should additionally be used. Thus, the *rpoB’* gene sequence of strain F20-122^T^ (611 bp) was also obtained and compared with those of the most closely related species. The phylogenetic tree analysis based on this gene by using the neighbor-joining algorithm showed that strain F20-122^T^ also clustered with the three species of the genus *Natronomonas* (Figure 2) but constituted a monophyletic branch clearly separated from those of the already described species, supporting its condition of a new taxon. Again, the topology of the tree constructed using the maximum-likelihood algorithm was in concordance with the neighbor-joining one.

**Figure 3:**
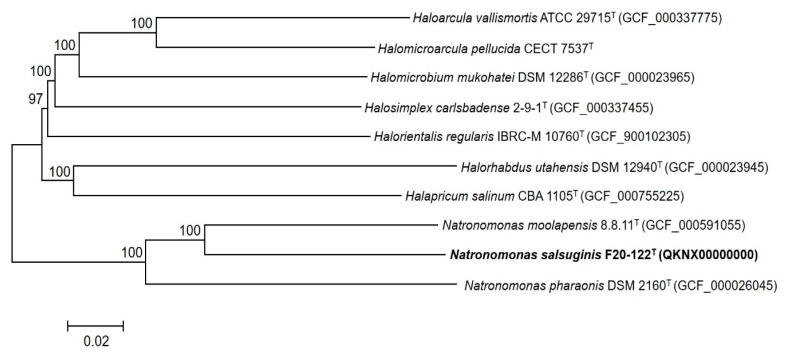
Phylogenomic tree reconstruction based on the core orthologous genes of strain F20-122^T^ and related species based on the neighbor-joining algorithm. This tree was obtained after the alignment of 319 shared orthologous single-copy genes of these genomes. Bootstrap values higher than 70% are indicated at branch points. Bar, 0.02 substitutions per nucleotide position.

In addition, a reconstruction of the phylogenetic core genome analysis was performed. The phylogenomic tree reconstruction (Figure 3), based on the alignment of 319 single-copy orthologous genes shared by all strains, revealed again that strain F20-122^T^ formed a clade with *Natronomonas pharaonis* DSM 2160^T^ and *Natronomonas moolapensis* 8.8.11^T^ with a bootstrap value of 100% but clustered in a different branch well separated from them with a bootstrap value of 100%, reinforcing its condition of a new taxon within the genus *Natronomonas*.

In order to confirm if strain F20-122^T^ would constitute a new species within the genus *Natronomonas*, the genomic parameters ANI and in silico DDH were estimated. The OrthoANI values between strain F20-122^T^, *Natronomonas pharaonis* DSM 2160^T^, and *Natronomonas moolapensis* 8.8.11^T^ were 75.9% and 79.8%, respectively (Table 1), while the in silico DDH values between these strains were 21.3 and 23.5%, respectively (Table 1). All these percentages were lower than 95–96% and 70%, respectively, which are the defined cut-off limits for species delineation [71,72,73,74,75], and confirm that strain F20-122^T^ is genotypically distinct to any previously described species and should be assigned to a different species.

On the basis of the evidence obtained from the phylogenetic and phylogenomic analyses and the ANI and in silico DDH parameters, strain F20-122^T^ should be considered as a novel species within the genus *Natronomonas*, for which the name *Natronomonas salsuginis* sp. nov. is proposed.

### 3.2. Phenotypic Characterization

To describe strain F20-122^T^ as a new species, the complete phenotypic characterization of this strain was carried out and compared with that of *Natronomonas moolapensis* CECT 7526^T^. Cells of strain F20-122^T^ were small coccobacilli shaped with a size of 1.0 µm (width) by 1.2–2.5 µm (length) (Figure S1). Cells of the new isolate F20-122^T^ were nonmotile and Gram-stain-negative. Colonies were circular, entire, pink pigmented, and 0.2–0.3 mm in diameter on R2A25% medium after 14 days of incubation at 37 °C. Strain F20-122^T^ is an extremely halophilic archaeon able to grow in media with 10%–30% (*w/v*) total salts, with optimal growth at 25% (*w*/*v*) total salts. It is not able to grow in the absence of salts. The pH range for growth was 6.5–9.0, showing the optimal growth at pH 8.0. Cells were able to grow from 25 to 50 °C, with the optimal growth at 37 °C. Catalase and oxidase activities were absent in strain F20-122^T^. The methyl red test was positive for the new isolate. Gelatin, starch, and aesculin were not hydrolyzed by strain F20-122^T^. Nitrate and nitrite were reduced. Other features of strain F20-122^T^ and the differential characteristics between strain F20-122^T^ and *Natronomonas moolapensis* CECT 7526^T^ are given in the species description and Table 2.

### 3.3. Chemotaxonomic Characterization

For the chemotaxonomic characterization, the total lipids of strain F20-122^T^ were extracted and compared with those from closely related neighbors, *Natronomonas pharaonis* CECT 4578^T^ and *Natronomonas moolapensis* CECT 7526^T^, and the reference strains *Halobacterium salinarum* DSM 3754^T^ and *Halorubrum saccharovorum* DSM 1137^T^. The HPTLC of the polar lipids (Figure S2) revealed that the polar lipid profile of strain F20-122^T^ consisted of phosphatidylglycerol phosphate methyl ester (PGP-Me) and phosphatidylglycerol (PG), both derived from C_20_C_20_ and C_20_C_25_ archaeol, and phosphatidylglycerol sulfate (PGS) as major lipids. Traces of biphosphatidylglycerol (BPG), minor phospholipids, and unidentified glycolipids were also detected. With the exception of the haloalkaliphilic species *Natronomonas pharaonis* CECT 4578^T^, the profile of polar lipids of strain F20-122^T^ shares the major lipids described for all species of *Natronomonas*; the presence of double chain length C_20_C_20_ and C_20_C_25_ derived from biphosphatidylglycerol (BPG) is not typically found on neutrophilic haloarchaeal species. This profile shows the divergence and variability of the lipidic membrane composition; in fact, this genus includes alkaliphilic and neutrophilic species, suggesting the adaptability of these strains to thrive in environments with pH fluctuations.

### 3.4. Genomic Characteristics

The draft genome sequence of strain F20-122^T^ was obtained and compared with those of the type species of the genus, *Natronomonas pharaonis* DSM 2160^T^, and the closest related species, *Natronomonas moolapensis* 8.8.11^T^. The main features of these genomes are shown in Table 3. The draft genome of strain F20-122^T^ was de novo assembled in a total of 13 contigs. The sequencing coverage depth of the entire genome was 531X with a N50 value of 675769 bp. This genome sequence is in accordance with the minimal standards for the use of genome data for the taxonomy of prokaryotes [76]. The genome size of strain F20-122^T^ was 2.9 Mb, identical to the genome of *Natronomonas moolapensis* 8.8.11^T^. The G+C content (63.2 mol%) and the number of rRNAs (3) and tRNAs (44) were also similar to those of the other *Natronomonas* species. Additional genomic characteristics are detailed in Table 3. 

Furthermore, a Venn diagram was constructed (Figure S3) showing the number of shared genes between strain F20-122^T^, *Natronomonas pharaonis* DSM 2160^T^, and *Natronomonas moolapensis* 8.8.11^T^.

### 3.5. Rhodopsin Analysis

Rhodopsins are photoreactive proteins first discovered in *Halobacterium salinarum* [77] with two different functions: light-driven ion transport (H^+^ pump bacteriorhodopsin [78], Cl^-^ pump halorhodopsin [79]) and phototaxis (sensory rhodopsins I and II [80,81]). Later on, distinct rhodopsins have also been identified in other organisms of the three domains of life, such as *Natronomonas pharaonis*, which has been a long-term target of study as a model organism for rhodopsin analyses due to its facile expression and purification in addition to other advantageous properties [82,83,84,85,86,87].

**Figure 4:**
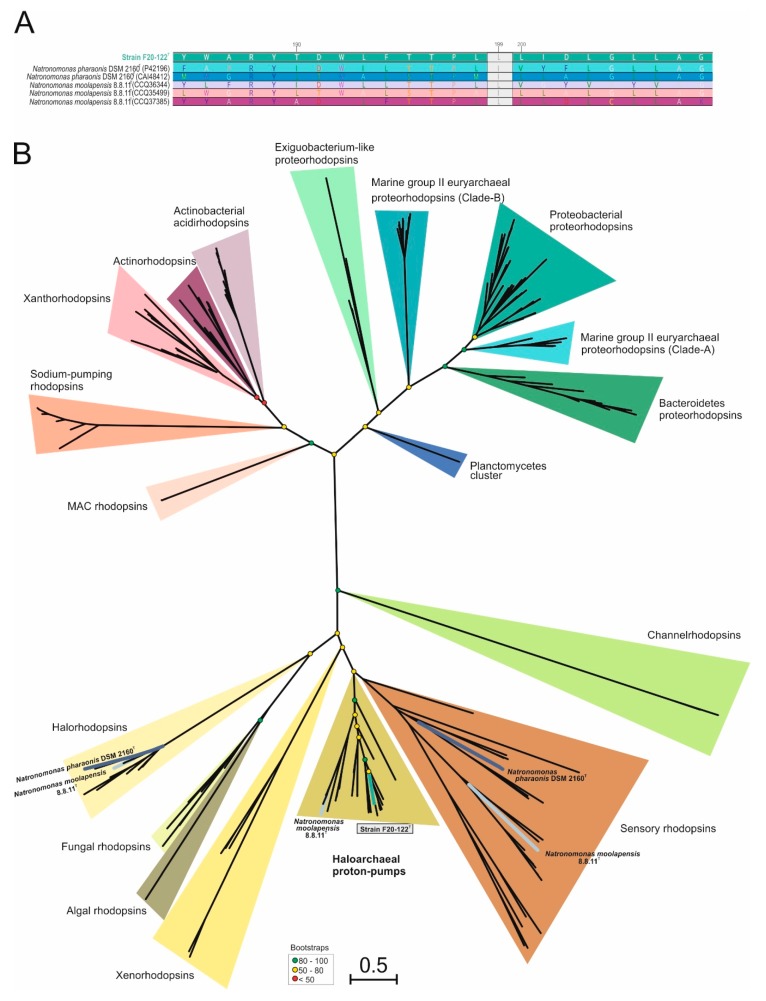
(**A**) Alignment comparison of rhodopsin sequences from species of the genus *Natronomonas*. Strain F20-122^T^ is highlighted in boldface. The green box exhibits the position 199 of the rhodopsin alignment, where the leucine (L) indicates a green absorption. (**B**) Maximum-likelihood phylogenetic tree based on 223 rhodopsin sequences. Colored branches were used to highlight rhodopsin sequences belonging to *Natronomonas* species. Bootstrap values on nodes are indicated by colored circles. Bar, 0.5 substitutions per nucleotide position.

Considering the affiliation of strain F20-122^T^ with the genus *Natronomonas*, the presence of rhodopsin-like sequences on the genomes of strain F20-122^T^, *Natronomonas moolapensis* 8.8.11^T^, and *Natronomonas pharaonis* DSM 2160^T^ was determined in detail (Figure 4). While halorhodopsins and sensory rhodopsins were found in *Natronomonas pharaonis* [88,89] and *Natronomonas moolapensis*, haloarchaeal proton pumps were identified as the unique rhodopsin sequences for strain F20-122^T^ (Figure 4). In addition, *Natronomonas moolapensis* also presented haloarchaeal proton pump rhodopsins, although situated in a different branch from strain F20-122^T^ (Figure 4B). The presence of haloarchaeal proton pumps for strain F20-122^T^ indicate a light-mediated ATP synthesis. These results are in accordance with previous metagenomic studies on hypersaline systems [30,90] which brought to light the existence of a large number of rhodopsin coding genes, suggesting that light is widely used in these extreme habitats.

On the other side, depending on the major absorption light wavelength, rhodopsins could be sorted as “green-absorbing” or “blue-absorbing”. This preference is determined by a single amino acid residue [91]. The rhodopsin-like sequence alignment identified in strain F20-122^T^ and other species of *Natronomonas* showed a leucine (L) amino acid residue in this position (Figure 4A), thus indicating a green spectrum absorption.

## 4. Conclusions

During the course of studies at different hypersaline environments located in the southwestern coast of Spain, a new extremely halophilic archaeon, strain F20-122^T^, phylogenetically related to the genus *Natronomonas* was isolated in pure culture and characterized using both new genomic and classical taxonomic methods in order to determine its precise affiliation. On the basis of this polyphasic taxogenomic study, it is concluded that strain F20-122^T^ constitutes a new species within the genus *Natronomonas*, for which the name *Natronomonas salsuginis* sp. nov. is proposed, the description of which is given below. Rhodopsin-encoding genes on the genome of this new haloarchaeon demonstrated the presence of proton pumps and the capacity for light-mediated ATP synthesis.

### Description of Natronomonas salsuginis sp. nov.

*Natronomonas salsuginis* (sal.su’gi.nis. L. gen. n. *salsuginis* of brackish water, pertaining to the salty water).

Cells are Gram-stain-negative, nonmotile, coccoid, and 1.0 × 1.2–2.5 µm. Does not grow anaerobically with L-arginine, DMSO, or potassium nitrate. Colonies are circular, entire, pink pigmented, and 0.2–0.3 mm in diameter on R2A25% medium after 14 days of incubation at 37 °C. Extremely halophilic, able to grow in media with 10%–30% (*w/v*) salts, with optimal growth at 25% (*w/v*) salts. No growth occurs in the absence of NaCl. Able to grow in the pH range of 6.5–9.0 and from 25 to 50 °C, with optimal growth at pH 8.0 and at 37 °C. Catalase and oxidase negative. Gelatin, starch, and aesculin are not hydrolyzed. Nitrate and nitrite are reduced. Urease and H_2_S production are negative. Simmons’ citrate and Voges–Proskauer tests are negative. Methyl red test is positive. Indole is not produced. Acid is produced from d-arabinose, d-fructose, d-glucose, and d-xylose but not from d-amygdalin, d,l-ethionine, glycerol, or d-sucrose. Starch, d-arabinose, d-cellobiose, fructose, d-glucose, lactose, maltose, d-mannose, l-raffinose, ribose, sucrose, d-trehalose, d-xylose, d-melezitose, butanol, dulcitol, ethanol, glycerol, d-mannitol, propanol, d-sorbitol, xylitol, methanol, benzoate, citrate, propionate, succinate, valerate, malate, pyruvate, or tartrate were not used as sole carbon and energy source. l-alanine, l-ornithine, l-glycine, l-lysine, l-threonine, and l-valine are used as sole carbon, nitrogen, and energy source but not L-arginine, asparagine, aspartic acid, l-cysteine, phenylalanine, glutamine, l-methionine, L-serine, tryptophan, and isoleucine. The major polar lipids are phosphatidylglycerol (PG), phosphatidylglycerol phosphate methyl ester (PGP-Me), and phosphatidylglycerol sulfated (PGS). Traces of biphosphatidylglycerol (BPG), other minor phospholipids, and unidentified glycolipids were also present. The DNA G+C content is 63.2 mol% (genome).

The type strain is F20-122^T^ (= CCM 8891^T^ = CECT 9564^T^ = JCM 33320^T^), isolated from a marine saltern located in Isla Bacuta, Huelva, Spain.

The GenBank/EMBL/DDBJ accession number for the 16S rRNA and *rpoB’* gene sequences of *Natronomonas salsuginis* F20-122^T^ are MH424601 and MH454090, respectively, and that of the complete genome is QKNX00000000.

## Figures and Tables

**Table 1:** Matrix of in silico DNA–DNA hybridization (DDH) and Ortho average nucleotide identity (OrthoANI) percentages between genomes of strain F20-122^T^ (showed in bold), species of the genus *Natronomonas*, and other related genera.

	ANI	1	2	3	4	5	6	7	8	9
In silico DDH										
**1**			**75.9**	**79.8**	**71.8**	**71.3**	**72.5**	**71.6**	**72.7**	**72.6**
**2**		**21.3**		76.5	71.5	71.2	72.1	71.6	72.2	72.4
**3**		**23.5**	21.5		72.2	71.9	72.9	71.9	72.6	72.8
**4**		**19.2**	19.5	19.6		73.8	74.0	72.9	74.0	73.9
**5**		**18.6**	19.4	19.1	19.8		73.1	72.1	73.3	73.0
**6**		**19.3**	19.8	19.4	20.2	19.8		74.8	75.1	74.5
**7**		**19.5**	19.4	19.7	19.8	19.3	20.6		73.9	73.3
**8**		**20.0**	19.7	20.0	20.4	19.9	20.9	20.7		74.6
**9**		**19.8**	20.1	20.5	20.5	20.0	20.5	20.3	20.6	

Strains: 1, Strain F20-122^T^ (QKNX00000000); 2, *Natronomonas pharaonis* DSM 2160^T^ (GCF_000026045); 3, *Natronomonas moolapensis* 8.8.11^T^ (GCF_000591055); 4, *Halapricum salinum* CBA 1105^T^ (GCF_000755225); 5, *Halorhabdus utahensis* DSM 12940^T^ (GCF_000023945); 6, *Halomicrobium mukohatei* DSM 12286^T^ (GCF_000023965); 7, *Haloarcula vallismortis* ATCC 29715^T^ (GCF_000337775); 8, *Halosimplex carlsbadense* 2-9-1^T^ (GCF_000337455); 9, *Halorientalis regularis* IBRC-M 10760^T^ (GCF_900102305).

**Table 2:** Differential characteristics of strain F20-122^T^ and the type strain of the most closely related species of the genus, *Natronomonas moolapensis* CECT 7526^T^.

Characteristics	1	2
Morphology	Coccoid	Rods or pleomorphic *
Motility	-	+ *
Cell size (µm)	1.0 × 1.2–2.5	0.7 × 1.7 *
Colony size (mm)	0.2–0.3	0.5–1.0
Colony pigmentation	Pink	Red *
NaCl range (optimum) (%, *w/v*)	10–30 (25)	14–36 * (18–20 *)
Temperature range for growth (optimum) (°C)	25–50 (37)	25–45 * (45 *)
pH range (optimum)	6.5–9.0 (8.0)	5.5–8.5 * (7.0–7.5 *)
Utilization as sole carbon and energy source of:		
Butanol	-	+
Ethanol	-	+
D-Glucose	-	+
Glycerol	-	+
Propanol	-	+
Salicin	-	+
Isoleucine	-	+
DNA G+C content (mol%, genome)	63.2	64.5

Strains: 1, F20-122^T^; 2, *Natronomonas moolapensis* CECT 7526^T^. All data from this study, except * which were obtained from the original description [5]. +, Positive; -, negative.

**Table 3:** General features of the genomes of strain F20-122^T^ and closely related species of the genus *Natronomonas* used in this study.

Feature	Strain F20-122^T^	*Natronomonas pharaonis* DSM 2160^T^	*Natronomonas moolapensis* 8.8.11^T^
Size (Mb)	2.9	2.6	2.9
Contigs	13	1	1
Completeness (%)	97.7	99.7	99.5
G+C (mol%)	63.2	63.4	64.5
N50 (bp)	675769	2595221	2912573
rRNA	3	3	3
tRNA	43	44	47
Accession number	QKNX00000000	GCF_000026045	GCF_000591055

## Supplementary Materials

The following are available online at https://www.mdpi.com/2076-2607/8/4/605/s1.
