# Supplementary material for: Natronomonas salsuginis sp. nov., a New Inhabitant of a Marine Solar Saltern
Source: Microorganisms. 2020 Apr 21;8(4):605. doi: 10.3390/microorganisms8040605 (PMC7232251; doi:10.3390/microorganisms8040605)
Supplement: Supplementary file 1 [file microorganisms-08-00605-s001.pdf]

microorganisms

Supplementary Material

Natronomonas salsuginis sp. nov., a New Inhabitant of a Marine Solar Saltern

Ana Durán-Viseras, Cristina Sánchez-Porro and Antonio Ventosa*

Department of Microbiology and Parasitology, Faculty of Pharmacy, University of Sevilla, 41012 Sevilla, Spain; anaduran@us.es (A.D.), sanpor@us.es (C.S.-P.), ventosa@us.es (A.V.)

* Correspondence: ventosa@us.es; Tel.: +34-954556765

Received: date; Accepted: date; Published: date

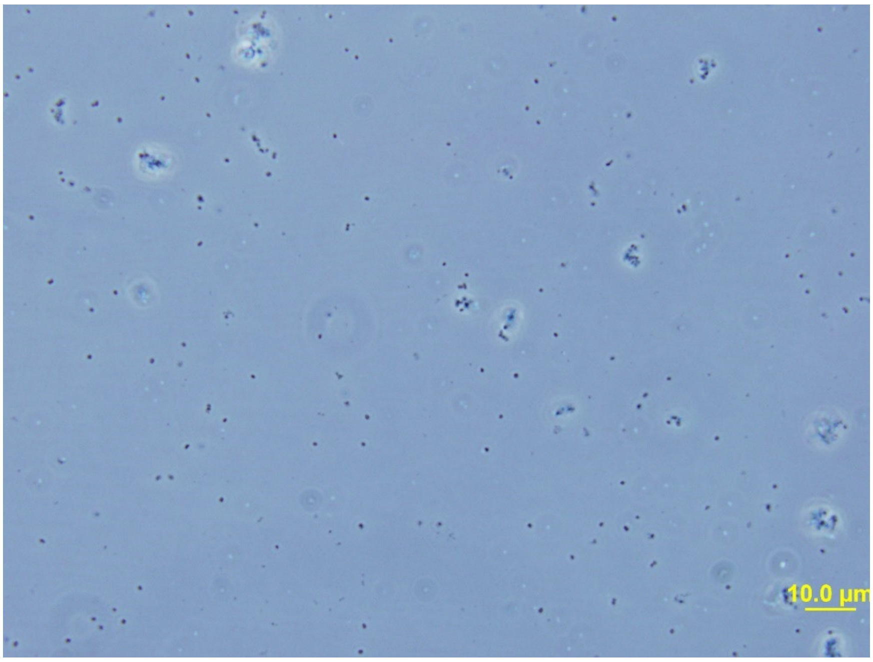

Figure S1. Phase-contrast photomicrograph of cells of strain F20-122^T cultured in liquid medium under optimal conditions. Scale bar, 10 µm.

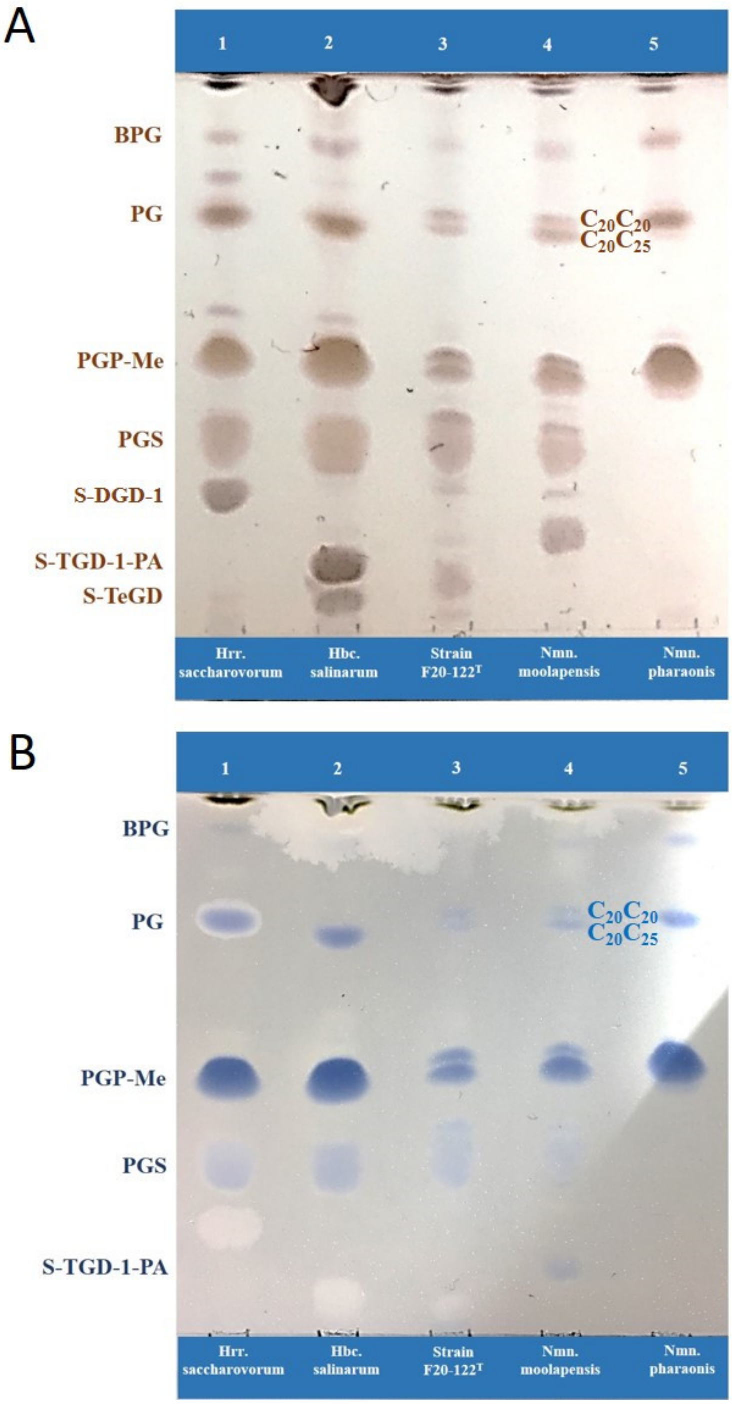

Figure S2. High performance thin layer chromatography (HPTLC) of polar lipids extracted from strain F20-122^{T} and some other haloarchaeal species. A) The plate was revealed with sulfuric acid 5 % in water, followed charred by heating at 160 °C. B) The plate was revealed with molybdenum blue spray reagent. Lanes: 1, *Halorubrum saccharovorum* DSM 1137^{T}; 2, *Halobacterium salinarum* DSM 3754^{T}; 3, Strain F20-122^{T}; 4, *Natronomonas moolapensis* CECT 7526^{T}; 5, *Natronomonas pharaonis* CECT 4578^{T}.

Abbreviations: BPG, biphosphatidylglycerol; PG, phosphatidylglycerol; PGP-Me, phosphatidylglycerol phosphate methyl ester; PGS, phosphatidylglycerol sulfate; S-DGD-1, sulfated diglycosyl diether; S-TGD-1-PA, sulfated triglycosyl diphytanyl archaeol ester linked to phosphatidic acid; S-TeGD, sulfated tetraglycosyl diether.

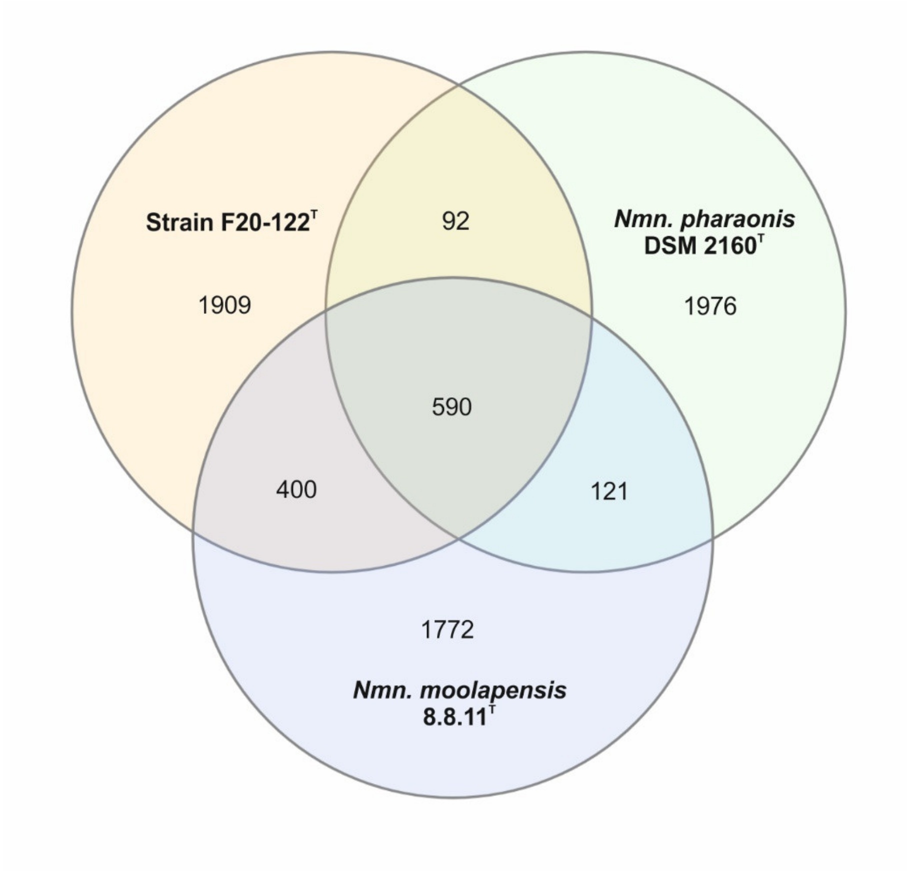

Figure S3. Venn diagram showing the number of genes shared between the genome of strain F20-122^T and closest related species *Natronomonas pharaonis* DSM 2160^T and *Natronomonas moolapensis* 8.8.11^T.An all-versus-all BLAST search and 70 % nucleotide identity was used for comparisons of all predicted protein-coding genes annotated from each genome.

© 2020 by the authors. Submitted for possible open access publication under the terms and conditions of the Creative Commons Attribution (CC BY) license (http://creativecommons.org/licenses/by/4.0/).
